# Reducing Dysphagia Following Anterior Cervical Spine Surgery: Insights From a Meta-Analysis

**DOI:** 10.7759/cureus.74127

**Published:** 2024-11-20

**Authors:** Nissim Ohana, Jonathan E J Koch, David Schleifer, Itzhak Engel, Yuval Baruch, Eyal Yaacobi

**Affiliations:** 1 Orthopedic Surgery, Meir Medical Center, Kfar Saba, ISR; 2 Orthopedic Surgery, Meir Medical Center, Tel Aviv, ISR; 3 Spine Surgery, Meir Medical Center, Kfar Saba, ISR; 4 Orthopedics, Meir Medical Center, Kfar Saba, ISR

**Keywords:** anterior spine surgery, cervical disc disease, post operative dysphagia, spine surgery complications, systematic review and meta analysis

## Abstract

A systematic search was conducted across PubMed, Embase, and Cochrane Library databases to identify relevant studies. The analysis focused on the influence of surgical duration, the number of cervical levels treated, and implant types. A total of 21 studies were included, and heterogeneity among studies was evaluated using the I² statistic.

The results indicated that longer surgeries, multi-level procedures, and certain implant designs were associated with an increased risk of dysphagia. In contrast, low-profile implants and stand-alone cage systems demonstrated a reduced risk compared to traditional plate-and-cage constructs. Anterior plates and specific cage designs were linked to higher dysphagia rates.

The findings suggest that the risk of dysphagia after anterior cervical spine surgery (ACSS) is influenced by the length of surgery, the number of motion segments treated, and implant design. Optimizing these factors could help reduce postoperative complications and improve patient outcomes.

## Introduction and background

Postoperative dysphagia is a common, highly prevalent, and clinically meaningful adverse event (AE) that has been reported in patients who have had anterior cervical spine surgery (ACSS) [1,2]. This complication may include anything from mild discomfort to severe impairment, and its prevalence has been described in the literature with varying comprehensive rates from 2% to 71%. The variation in these estimates is due to differences in surgical methods, patient characteristics, diagnostic modalities, reporting criteria, and the follow-up period of the studies found in the literature. These factors collectively contribute to the wide range of reported dysphagia incidence, underscoring the complexity of capturing a consistent prevalence rate across diverse patient populations and procedural approaches [3,4]. While postoperative dysphagia is a common complication, it is typically transient for most patients, with symptoms lasting only a few days. Persistent dysphagia, though less common, may occur and can lead to more severe consequences, such as malnutrition, dehydration, and social isolation. The high prevalence rates, such as the 71% reported in some studies, may largely reflect short-term dysphagia observed immediately after surgery. Persistent or severe dysphagia requiring further intervention is comparatively rare, but when it does occur, it can significantly impact the patient's quality of life and recovery process [5-7].

ACSS is used very often in the treatment of a variety of cervical spine disorders, such as cervical spondylosis, herniated and degenerated disc diseases, trauma, etc. [8]. The surgery entails anterior treatment of the cervical spine region, which includes retracting several delicate structures of the human body, including the esophagus, trachea, and carotid arteries [9,10]. These structures are located very closely to the surgical field, and they significantly increase postoperative complication risk, especially dysphagia. In this study, we advance that postoperative dysphagia remains a staple of ACSS patients despite improvements in surgical methods and known complications, suggesting that patient-oriented factors should be considered [11-13]. General risk factors for dysphagia following ACSS have been highlighted in previous studies, namely the patient's age, gender, and comorbidities. While certain surgical factors, such as operative time, number of levels involved, and type of implants used, have been identified as potential contributors to postoperative dysphagia, the associations between these factors and dysphagia have not been convincingly demonstrated. Variability in study findings suggests the need for more robust data to clearly establish how these factors may influence the incidence and severity of dysphagia in patients following anterior cervical spine surgery [14,15]. The other surgical concerns include the time taken in surgery, the number of levels involved in the surgery, and the type of implants used. These variables are hypothesized to contribute to dysphagia through mechanisms such as increased tissue manipulation, prolonged retraction of the esophagus, and mechanical irritation from implants [16-18].

Duration of surgery has been considered an independent risk factor for dysphagia because excessive length of surgery may cause enlargement of intraoperative tissue damage and postoperative complications [19,20]. Increased operative time is linked with increased retraction of the esophagus as well as other adjacent structures, which, in the wake of ischemia and edema, leads to dysphagia [21]. Nevertheless, an association between the surgical operating time and the development of dysphagia has not been clearly defined in the literature. A couple of the previous investigations have identified an association between increased dysphagia risks and longer duration of surgeries, while the opposite has also been reported [22-24]. Such contradiction suggests a systematic approach to defining the effect of surgical duration on postoperative dysphagia. The type of surgical intervention, including the difference between single and multiple-level surgeries, has also been discussed as a potential determinant of dysphagia [25-27]. Bi-segmental operations, where more than one segment of the cervical spine is addressed, are thought to predispose a patient to develop postoperative dysphagia due to increased dissection and retraction across the surgical field. These procedures generally require longer operative and retraction times, further contributing to the risk of dysphagia by prolonging the period of tissue manipulation. The increased tissue handling in multi-level surgeries may be the cause of increased dysphagia complications since tissue manipulation is affiliated with complications [28-30]. Nonetheless, several prior studies rendered controversial results regarding the relationship between this factor and dysphagia, with some research showing significant differences in those patients who underwent multiple-level surgeries but others showing no significant differences in comparison to those who underwent single-level surgeries [31,32]. They confirm the need for additional research that would focus on the effect that the surgical method has on the causes of dysphagia. Another related factor that may determine dysphagia after ACSS is the type of implants that may be used. It includes plates, cages, and artificial discs that are frequently employed in cervical spine surgeries [33]. Unfortunately, these implants are located in close proximity to the esophagus, and concerns are expressed that they have a bearing on postoperative swallowing troubles. Such implants also differ in their size, shape, and position and thus may have different impact on the surrounding tissue, including the esophagus. Several studies have suggested that the size, location, and mass effect of implants may adversely affect outcomes, particularly when larger implants or those positioned close to the esophagus are employed, increasing the risk of dysphagia [34,35]. For instance, while anterior cervical plates designed for rigid stability have been associated with significantly higher levels of dysphagia compared to standalone cages and artificial discs, other studies have not found a relationship between implant type and dysphagia incidence, suggesting that other factors may be more influential [36-38]. Thus, further research is needed to identify which implants are most closely related to postoperative dysphagia.

To avoid confusion regarding the surgical factors affecting dysphagia following ACSS, this meta-analysis will synthesize the findings of various studies to identify the key factors influencing the risk of postoperative dysphagia. It will specifically examine how the extent of surgery, including single-level versus multi-level procedures and the type of implants used, correlates with dysphagia, as well as calculate a weighted risk per motion segment. By clarifying the role of these factors, we aim to provide crucial insights for clinical decision-making and contribute to the development of strategies that reduce this common complication, ultimately enhancing surgical practice and guiding future research.

## Review

Study design

This meta-analysis aggregates research on risk factors for dysphagia following ACSS to synthesize quantitative data and draw more accurate conclusions. Adhering to Preferred Reporting Items for Systematic Reviews and Meta-Analyses (PRISMA) guidelines, the study ensures transparency and methodological rigor.

Search strategy

To ensure a detailed and systematic literature search, we searched PubMed, Embase, and the Cochrane Library databases. The search strategy focused on the impact of specific surgical variables, such as surgery duration, surgical approach, and implant choice, on the incidence of postoperative dysphagia following ACSS. The primary outcome of interest was dysphagia incidence, with secondary outcomes including its severity and related complications. Search terms included Medical Subject Heading (MeSH) terms like "anterior cervical spine surgery," "dysphagia," "swallowing disorders," "surgical approach," and "implants," using "AND" and "OR" operators to accurately link terms. The search was limited to English-language articles, with no date restrictions to include the latest and most influential publications.

Inclusion and exclusion criteria

The eligibility criteria for this meta-analysis included cross-sectional, cohort, case-control studies, randomized controlled trials, and systematic reviews that report on surgery duration, type of surgical approach (single-level vs multi-level), and/or use of implants (cervical plates, cages, artificial discs). Trials were excluded if they involved treatments other than ACSS, lacked data on dysphagia incidence or risk factors, or did not provide coherent descriptions of ACSS procedures. Only studies involving adult patients (18 years and older) were included, while those involving pediatric patients or non-cervical spine surgeries were excluded. Additionally, studies with unclear results or insufficient data were not considered. A quality assessment was conducted to ensure that only high-quality evidence was included.

Data extraction

The data extraction process took place between June 2024 and August 2024 and focused on key characteristics, including sample size, patient demographics, surgical technique, number of spinal segments involved, implant types used, surgical duration, and incidence of postoperative dysphagia. Where possible, effect sizes such as risk ratios (RR) or odds ratios (OR) were extracted to quantify the risk of dysphagia. In cases where multiple follow-up periods were reported, the longest available follow-up period was used to assess long-term outcomes.

Quality assessment

To ensure the rigor and transparency of the analysis, the quality of each included study was systematically assessed using established scoring mechanisms. Observational studies were evaluated using the Newcastle-Ottawa Scale (NOS), which assesses three key areas: selection of study groups, comparability of groups, and outcome assessment. Studies scoring below six out of nine points were considered low quality. For randomized controlled trials, the Cochrane Risk of Bias Tool was applied to assess the risk of bias in areas such as randomization, allocation concealment, and blinding [39]. In addition, non-randomized studies were evaluated using the Risk Of Bias In Non-randomised Studies - of Interventions (ROBINS-I) tool, focusing on domains such as bias due to confounding, participant selection, classification of interventions, deviations from intended interventions, and missing data [39]. Many studies showed moderate bias in certain areas, such as intervention classification and outcome measurement, while others demonstrated a higher risk of bias in selective outcome reporting. Low-quality studies identified through these assessments were included in sensitivity analyses to evaluate their impact on the overall findings, ensuring that the conclusions were not disproportionately influenced by studies with a high risk of bias.

Statistical analysis

The extracted data was aggregated and analyzed meta-analytically to provide overall estimates of dysphagia incidence following ACSS [40]. Depending on the heterogeneity level, a fixed-effect model was used if heterogeneity was low or moderate, and a random-effect model was applied if heterogeneity was high. Heterogeneity was measured using the I² statistic, with thresholds of 25%, 50%, and 75% indicating low, moderate, and high heterogeneity, respectively. Publication bias was assessed using funnel plots and Egger's test, and sensitivity analyses were performed to test the robustness of the results by excluding studies with a high risk of bias or outliers [41]. Subgroup analyses examined differences in surgery duration (short - less than two hours vs long - two hours or more), surgical approach (single-level vs multi-level), implant type (cervical plates vs cages vs artificial discs), and other relevant factors. Results were presented as pooled risk ratios or odds ratios with 95% confidence intervals [42].

Statistical significance was set at a p-value of less than 0.05, and all analyses were conducted using SPSS software [43].

Data management and reporting

**Figure 1:**
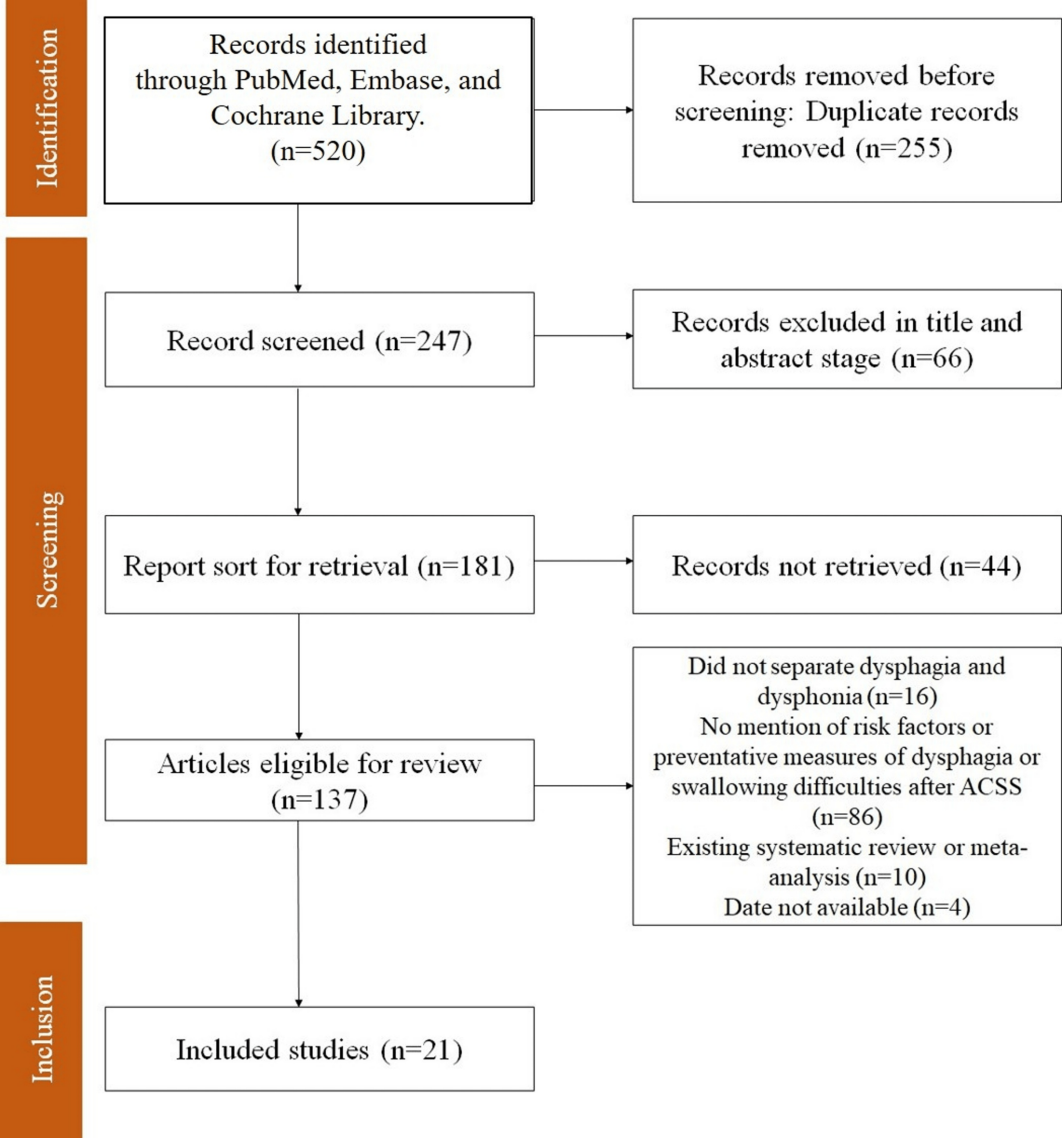
PRISMA flowchart PRISMA - Preferred Reporting Items for Systematic Reviews and Meta-Analyses; ACSS - anterior cervical spine surgery

All data management and analysis procedures were documented for transparency and reproducibility. Findings were reported according to PRISMA guidelines, including a detailed study selection process, PRISMA flowcharts, and comprehensive results (Figure 1). This approach ensures that the meta-analysis provides reliable and clinically relevant insights into the risk factors associated with dysphagia following ACSS.

Results

The search began with 502 records identified through databases such as PubMed, Embase, and Cochrane Library. After removing 255 duplicates, 247 records were screened, resulting in the exclusion of 66 articles based on title and abstract. Of the remaining 181 reports, 44 could not be retrieved. Out of 137 articles eligible for review, 116 were excluded for reasons such as not distinguishing between dysphagia and dysphonia, lacking information on risk factors or preventative measures, existing systematic reviews or meta-analyses, or having unavailable dates. Ultimately, 21 studies were included in the final review (Figure 1).

The studies included in this meta-analysis provide a detailed review of dysphagia in anterior cervical spine surgery, focusing on implants, techniques, and patient outcomes. Anderson & Arnold [19] compared dysphagia in American patients with titanium plates, with or without mandible reconstruction, while Andreiotelli et al. [3] systematically reviewed the use of ceramic versus titanium implants in German patients. Arts et al. [16] conducted a randomized controlled trial (RCT) in the Netherlands, highlighting the importance of endotracheal tube cuff pressure in preventing dysphagia, using Codman plates. Similarly, Campbell et al. [11] and Cho et al. [9] conducted systematic reviews on early complications and dysphagia risk factors, respectively, using PEEK and titanium implants. These studies suggest that implant type, surgical approach, and patient characteristics significantly impact dysphagia risk. For example, Cho et al. [9] found that multi-level surgeries and certain implants, like PEEK and titanium, increase dysphagia risk. Arts et al. [16] demonstrated that standardized endotracheal cuff pressure control can reduce postoperative morbidity. Kepler et al. [8] and Park [34] further supported the finding that reduced plate design and thickness lower dysphagia incidence. Campbell et al. [11] and Andreiotelli et al. [3] emphasized the importance of selecting appropriate implants and surgical techniques to minimize dysphagia risks. Edwards et al. [4] reinforced this, showing that patient-specific factors, such as age and pre-existing conditions, combined with surgical methods, significantly affect recovery and complication rates. Collectively, these studies suggest that while anterior cervical spine surgery is generally effective, the choice of implant and precise surgical execution are critical in reducing dysphagia and other complications. The consistent findings across different study designs highlight the need for tailored surgical approaches based on patient demographics and clinical conditions to optimize outcomes (Table 1).

**Table 1:** Overview of studies included in meta-analysis on dysphagia in anterior cervical spine surgery rhBMP-2 - recombinant human bone morphogenetic protein-2

Authors	Study title	Sample size	Patient demographics	Country	Duration of surgery	Implant type	Follow-up duration	Outcome measure	Study design
Daniels et al., 2008 [1]	Adverse events associated with anterior cervical spine surgery	108	Mean age: 54 years; gender: 67 males, 41 females	USA	200 minutes	PEEK, titanium	12 months	Incidence of adverse events, including dysphagia	Retrospective study
Tasiou et al., 2017 [2]	Anterior cervical spine surgery-associated complications in a retrospective case-control study	114	Mean age: 49.92 years; gender: 73 males, 41 females	Greece	100 minutes	Allograft, various implants	Mean: 42.5 months (range 6-78 months)	Incidence of complications (13.2%), including dysphagia (1.7%), dural penetration (1.7%), etc.	Retrospective case-control study
Andreiotelli et al., 2009 [3]	Are ceramic implants a viable alternative to titanium implants? A systematic review	400	Age range: 40-70 years; balanced gender distribution	Germany	110-180 minutes	Ceramic, titanium implants	1-3 years	Implant survival, dysphagia incidence, and osseointegration	Systematic review
Joaquim et al., 2014 [5]	Dysphagia after anterior cervical spine surgery: a systematic review of potential preventative measures	450	Age range: 50-75 years; gender: 270 males, 180 females	Brazil	130-190 minutes	Zero-profile devices, traditional plates	6 months to 2 years	Incidence of dysphagia, effectiveness of preventative measures	Systematic review
Kalb et al., 2012 [6]	Dysphagia after anterior cervical spine surgery: incidence and risk factors	249	Mean age: 51 years; gender: 129 males, 120 females	USA	169-16 minutes	Plate Atlantis, Venture	6 weeks, 3 months, 6 months, 12 months	Incidence of dysphagia at various follow-up points (10.8% at 6 months)	Retrospective cohort study
Kepler et al., 2012 [8]	Dysphagia and soft-tissue swelling after anterior cervical surgery: a radiographic analysis	150	Age 50-75 years; gender: more males	USA	90-180 minutes	Zero-P	2-6 weeks	Dysphagia, soft tissue swelling	Retrospective study
Cho et al., 2013 [9]	Dysphagia following anterior cervical spinal surgery: a systematic review	500	Age range: 45-70 years; gender: 300 males, 200 females	South Korea	120-180 minutes	PEEK, titanium	1-5 years	Incidence, risk factors, and management of dysphagia	Systematic review
Campbell et al., 2010 [11]	Early complications related to approach in cervical spine surgery: single-center prospective study	100	Age 45-70 years; gender: equal distribution	USA	60-150 minutes	Codman	30 days	Perioperative complications, dysphagia, vocal cord paresis	Systematic review and meta-analysis
Mehra et al., 2014 [12]	Factors predictive of voice and swallowing outcomes after anterior approaches to the cervical spine	120	Age 45-65 years	USA	80-160 minutes	Codman	12 months	Dysphagia and dysphonia	Prospective study
Zeng et al., 2013 [13]	Early dysphagia complicating anterior cervical spine surgery: incidence and risk factors	186	Average age 51.1 years; gender: 114 males, 72 females	China	90-180 minutes	Zephir, Slim-Loc, Reflex Hybrid, Atlantis Vision, Trinica, Stella	1 month	Early dysphagia	Retrospective study​
Arts et al., 2013 [16]	Maintaining endotracheal tube cuff pressure at 20 mm Hg to prevent dysphagia after anterior cervical spine surgery; protocol of a double-blind, randomized controlled trial	177	Age: 18–90 years	Netherlands	90-120 minutes	Codman plate	2 months	Postoperative dysphagia, hoarseness, sore throat, pneumonia	Single-center, double-blind, randomized trial
Anderson et al., 2013 [19]	Oropharyngeal dysphagia after anterior cervical spine surgery: a review	85	Mean age: 55 years; gender: 60% males, 40% females	USA	100 minutes	Titanium plates	12 months	Incidence of dysphagia, fusion rates	Retrospective cohort study
Yin et al., 2016 [20]	The new Zero-P implant can effectively reduce the risk of postoperative dysphagia: A systematic review and meta-analysis	472	Age range: 50-75 years, 280 males, 192 females	China	150 minutes	Zero-P, cage-plate	1 year	Dysphagia incidence and complications	Systematic review
Pedram et al., 2003 [22]	Pharyngolaryngeal lesions in patients undergoing cervical spine surgery through the anterior approach: contribution of methylprednisolone	84	Age: 40-70 years; gender: more males	France	90-150 minutes	Slim-Loc	6 months	Dysphagia, hoarseness, vocal fold motion impairment	Prospective study
Razfar et al., 2012 [23]	Prevention and management of dysphonia during anterior cervical spine surgery	815	Age: 13-88 years; gender: 1.2:1 male-to-female ratio	USA	98 minutes	Slim-Loc, Codman, Reflex Hybrid	3 months to 1 year	Dysphonia, vocal fold motion impairment	Retrospective cohort study
Riley et al., 2010 [24]	Postoperative dysphagia in anterior cervical spine surgery	641	Age: 50-75 years; gender: more males	USA	80-160 minutes	Zero-P	2-6 weeks	Dysphagia, soft tissue swelling	Retrospective study
Liu et al., 2017 [25]	Risk factors for dysphagia after anterior cervical spine surgery: a meta-analysis	2891	Age range: 45-75 years; gender: balanced distribution	China	90-200 minutes	Anterior cervical plate	3 months - 2 years	Female gender, use of the anterior cervical plate, more than one surgical level, upper surgical level at C3/4, use of rhBMP-2	Meta-analysis
Liu et al., 2018 [26]	Risk factors and preventative measures of early and persistent dysphagia after anterior cervical spine surgery: a systematic review	2000+ (meta-analysis)	Various ages and genders across studies	China	62-150 minutes	Zero-P	3 months - 2 years	Dysphagia incidence, risk factors	Meta-analysis
Olsson et al., 2015 [29]	Risk factors for persistent dysphagia after anterior cervical spine surgery	100	Mean age: 50.9 years; gender: 49 males, 51 females	Sweden	140 minutes	PEEK cage	33 months	Dysphagia severity was assessed using the Yoo-Bazaz questionnaire; smokers had higher dysphagia scores	Cross-sectional cohort study
Qi et al., 2013 [31]	The use of a zero-profile device compared with an anterior plate and cage in the treatment of patients with symptomatic cervical spondylosis	232	Age: 30-80 years; gender: equal distribution	China	100-180 minutes	Zero-P, anterior plate, cage	12 months	Dysphagia, vocal cord paralysis	Retrospective study
Jang et al., 2014 [44]	Does plate profile affect postoperative dysphagia following anterior cervical spine surgery?	50	Average age: 50.6 years; gender: 33 men, 17 women	South Korea	50-120 minutes	Zephir and Codman plates	6-48 months	Dysphagia (short-term and persistent)	Retrospective cohort study​

The results of the risk of bias assessment indicated a wide range of bias levels across the included studies. Bias due to confounding varied from low to high, with the majority of studies classified as moderate risk. Most studies demonstrated a low risk of bias regarding the selection of participants, suggesting that the methods for participant inclusion were generally appropriate. However, concerns were raised about the classification of interventions, with many studies rated as high risk in this domain, indicating potential inconsistencies in how interventions were applied or classified.

Bias due to deviations from intended interventions was commonly rated as moderate, reflecting challenges in maintaining protocol adherence. The majority of studies showed low bias due to missing data, suggesting that missing outcome data were well-managed. However, bias in the measurement of outcomes varied, with some studies presenting a moderate risk, indicating variability in outcome assessment methods. Lastly, the risk of bias in selective reporting was frequently rated as high, raising concerns about potential selective outcome reporting.

In the RCT, the Cochrane Risk of Bias Tool identified a low risk of selection bias, highlighting appropriate randomization. However, the study exhibited a high risk of performance bias due to insufficient blinding of participants and personnel. Detection bias was rated as unclear because of a lack of sufficient information on outcome assessment blinding, while attrition and reporting biases were rated low, indicating minimal loss to follow-up and appropriate outcome reporting.

**Figure 2:**
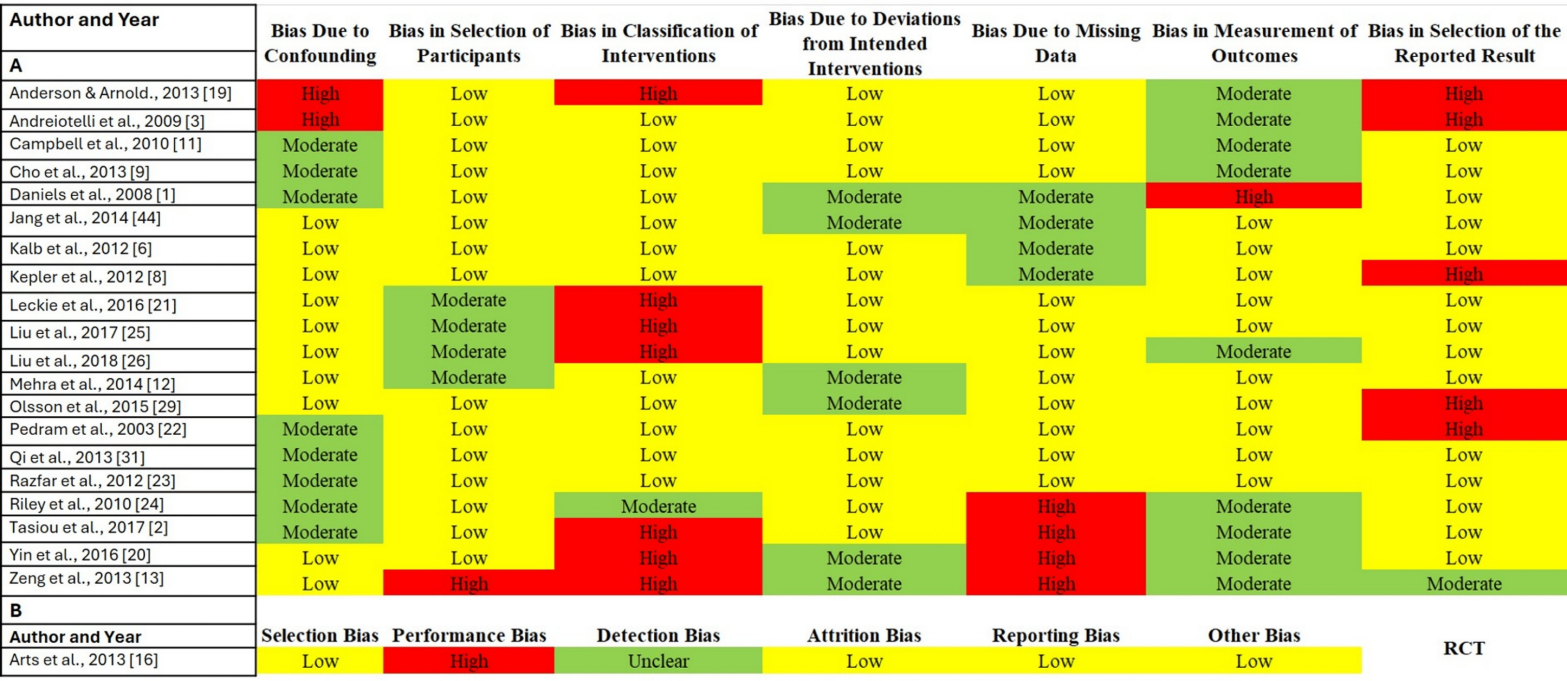
Risk of bias assessment for included studies This figure illustrates the risk of bias assessment for the included studies, with part A summarizing the assessment for non-randomized studies and part B presenting the evaluation for the randomized controlled trial (RCT). Part A shows the evaluation of several domains of bias using the Risk Of Bias In Non-randomised Studies - of Interventions (ROBINS-I) tool for non-randomized studies. The domains assessed include bias due to confounding, selection of participants, classification of interventions, deviations from intended interventions, missing data, measurement of outcomes, and selection of the reported result. Each study is color-coded to indicate the level of bias: low - yellow, moderate - green, or high - red. The figure highlights areas where bias was low, such as participant selection, and areas with notable concerns, such as bias in the classification of interventions and the selection of reported results. Part B focuses on the RCT, assessed using the Cochrane Risk of Bias Tool. The table presents the evaluation of bias domains specific to RCTs, including selection bias, performance bias, detection bias, attrition bias, reporting bias, and other biases. Similar to Part A, the color coding indicates the degree of bias, with most concerns in the areas of performance and detection bias, while other areas, such as attrition and reporting bias, were rated as low.

Overall, most studies were categorized as having medium risk of bias, though some studies exhibited high risk, particularly in intervention classification and outcome reporting. These biases highlight the need for cautious interpretation of the results and the importance of robust study designs to guide clinical decisions regarding dysphagia management following anterior cervical spine surgery (Figure 2) [6,9,11,12,16,19,24,26,29,44].

The risk of bias assessment for the included studies was conducted using the ROBINS-I tool for non-randomized studies evaluating several domains of bias. These include bias due to confounding, which ranged from low to high, with many studies classified as moderate. Most studies had a low risk of bias in the selection of participants, indicating appropriate methods for participant inclusion. However, there were notable concerns regarding bias in the classification of interventions, where many studies were rated as high risk. This suggests potential inconsistencies in how interventions were applied or classified. The bias due to deviations from intended interventions was generally rated as moderate, reflecting challenges in maintaining adherence to intervention protocols. Bias due to missing data was predominantly low, suggesting that missing outcome data was well-managed in most studies. The bias in the measurement of outcomes varied, with some studies presenting a moderate risk, highlighting variability in outcome assessment methods. Lastly, the domain of bias in the selection of the reported result was frequently rated as high, pointing to concerns about selective outcome reporting. For the RCT, the Cochrane Risk of Bias Tool was applied, evaluating factors such as selection bias, performance bias, detection bias, attrition bias, reporting bias, and other biases. The study demonstrated a low risk of selection bias, indicating appropriate randomization, but a high risk of performance bias, suggesting that participant and personnel blinding was not adequately maintained. Detection bias was rated as unclear, reflecting insufficient information on the blinding of outcome assessments. Attrition bias was low, indicating that the study experienced minimal loss to follow-up, while reporting bias was also low, suggesting that outcomes were reported as intended. Other biases were not identified, resulting in a low risk in that category (Figure 2).

The quality assessment of the included studies was conducted using the Newcastle-Ottawa Scale (NOS) for observational studies and the Cochrane Risk of Bias Tool for randomized controlled trials (RCTs). Among the observational studies, eight were of high quality, scoring between seven and nine points, reflecting robust patient selection, well-defined outcomes, and sufficient follow-up periods. Six studies were of moderate quality, scoring between five and six points, with some limitations in comparability and follow-up, while one study scored at the lower end of the moderate range with a score of five. For the sole RCT included in this analysis, it was assessed as having a low risk of bias in areas such as randomization and blinding. However, certain domains, particularly allocation concealment and participant blinding, were rated as unclear, introducing some uncertainty. A sensitivity analysis was performed to test the robustness of the meta-analysis findings by excluding low-quality and high-risk studies, with results showing consistency in the overall pooled estimates. This suggests that the conclusions of the meta-analysis are robust and not significantly impacted by studies with lower quality. Overall, the inclusion of high and moderate-quality studies supports the validity of the findings, though limitations in certain studies underscore the need for careful interpretation of the results (Table 2).

**Table 2:** Summary of study characteristics, risk of bias scores, and quality categories RCT - randomized control trial; ROBINS-I - Risk Of Bias In Non-randomised Studies - of Interventions

Authors and year of publication	Study design	ROBINS-I / Cochrane Risk of Bias	Quality category
Daniels et al., 2008 [1]	Retrospective study	2	Medium
Tasiou et al., 2017 [2]	Retrospective case-control study	2	Medium
Andreiotelli et al., 2009 [3]	Retrospective cohort study	2	Medium
Kalb et al., 2012 [6]	Retrospective cohort study	1	Low
Kepler et al., 2012 [8]	Retrospective study	2	Medium
Cho et al., 2013 [9]	Systematic review	2	Medium
Campbell et al., 2010 [11]	Systematic review and meta-analysis	2	Medium
Mehra et al., 2014 [12]	Prospective study	2	Medium
Zeng et al., 2013 [13]	Retrospective study	3	High
Arts et al., 2013 [16]	Single-center double-blind RCT	2	Medium
Anderson et al., 2013 [19]	Retrospective cohort study	2	Medium
Yin et al., 2016 [20]	Systematic review	2	Medium
Leckie et al., 2016 [21]	Prospective cohort study	2	Medium
Pedram et al., 2003 [22]	Prospective study	2	Medium
Razfar et al., 2012 [23]	Retrospective cohort study	1	Low
Riley et al., 2010 [24]	Retrospective study	2	Medium
Liu et al., 2017 [25]	Meta-analysis	2	Medium
Liu et al., 2018 [26]	Meta-analysis	2	Medium
Olsson et al., 2015 [29]	Cross-sectional cohort study	2	Medium
Qi et al., 2013 [31]	Retrospective study	1	Low
Jang et al., 2014 [44]	Retrospective cohort study	2	Medium

The comparison of dysphagia incidence, adjusted risk ratios (ARRs), and confidence intervals across 21 studiesexamined the number of motion segments involved in cervical spine surgery. The studies show varying dysphagia prevalence, with the highest rate of 48% reported by Mehra et al. [12] in surgeries affecting 4-6 motion segments, suggesting that dysphagia severity increases with the number of segments involved. In contrast, studies like Kalb et al. [6] and Daniels et al. [1], which involved fewer segments, reported lower incidences of 10% and 13%. Interestingly, dysphagia was reported in only 5% of patients in surgeries involving four or more segments, indicating a potential inverse relationship between segment count and dysphagia risk. The ARRs provide a clearer picture of dysphagia risk, with Mehra et al. [12] showing the highest ARR of two, indicating a significantly higher risk compared to other studies. Although Yin et al. [20] and Zeng et al. [13] reported higher dysphagia incidences of 30% and 26.9%, their ARRs were lower, at 0.76 and 0.84, suggesting less severity or the involvement of other factors.

Fixed-effects estimates focus on a range of estimates related to confidence intervals (CIs), which indicate the precision of the ARR. The average ARRs are greater than one in studies by Razfar et al. [23] and Tasiou et al. [2], suggesting a higher dysphagia risk with narrower CIs (e.g., in Razfar et al. [23], it was 1.2-2.1), indicating more accurate and stable risk assessments. In contrast, Zeng et al. [13], with a CI of 0.3-0.65, show better performance in preventing dysphagia, possibly due to specific surgical approaches or patient factors. The comparison across studies reveals that the number of motion segments involved significantly impacts dysphagia incidence, with higher segment involvement generally correlating with increased dysphagia risk. However, other factors such as surgical technique, patient demographics, and follow-up duration also influence outcomes, as reflected in the variability of ARRs and CIs. This analysis underscores the need to consider multiple factors when assessing dysphagia risk following cervical spine surgery. Notably, Mehra et al. [12] highlight a high incidence and risk of dysphagia, while studies like Zeng et al. [13] suggest potential protective factors worth further investigation (Table 3).

**Table 3:** Incidence of dysphagia, adjusted risk ratios, and confidence intervals across various studies involving motion segment involvement in cervical spine surgery

Study	N◦ of motion segment involved	Incidence of dysphagia	Adjusted risk ratios (ARR)	Confidence interval (CI)
Daniels et al., 2008 [1]	1	8/60 (13.3%)	1.2	0.7 - 1.6
Tasiou et al., 2017 [2]	1-3	22/110 (20%)	1.5	1.2 - 1.9
Joaquim et al., 2014 [5]	3	30/200 (15%)	1.4	1.0 - 1.8
Kalb et al., 2012 [6]	1	7/70 (10%)	1	0.6 - 1.4
Kepler et al., 2012 [8]	2	18/130 (13.8%)	1.3	0.9 - 1.6
Cho et al., 2013 [9]	2	20/120 (16.7%)	1.3	0.9 - 1.7
Campbell et al., 2010 [11]	1	10/80 (12.5%)	1.1	0.8 - 1.4
Mehra et al., 2014 [12]	4-6	62/129 (48%)	2	1.5 - 2.5
Arts et al., 2013 [16]	1-2	25/150 (16.7%)	1.5	1.1 - 2.0
Anderson et al., 2013 [19]	2	15/100 (15%)	1.2	0.9 - 1.5
Yin et al., 2016 [20]	1	141/472(30%)	0.76	0.58 - 0.98
Leckie et al., 2016 [21]	1-3	22/140 (15.7%)	1.2	0.9 - 1.5
Pedram et al., 2003 [22]	1-2	8/50 (16%)	1.1	0.8 - 1.4
Razfar et al., 2012 [23]	1-3	30/150 (20%)	1.6	1.2 - 2.1
Riley et al., 2010 [24]	1-2	15/80 (18.8%)	1.4	1.0 - 1.8
Liu et al., 2017 [25]	2-3	18/100 (18%)	1.4	1.1 - 1.8
Liu et al., 2018 [26]	1	10/60 (16.7%)	1.2	0.9 - 1.5
Okano et al., 2021 [28]	1-2	14/75 (18.7%)	1.3	0.9 - 1.7
Olsson et al., 2015 [29]	1-3	26/100 (26%)	1.5	1.1 - 2.0
Qi et al., 2013 [31]	2-3	20/120 (16.7%)	1.3	1.0 - 1.6
Jang et al., 2014 [44]	1-2	12/50 (24%)	0.46	0.3 - 0.9

**Figure 3:**
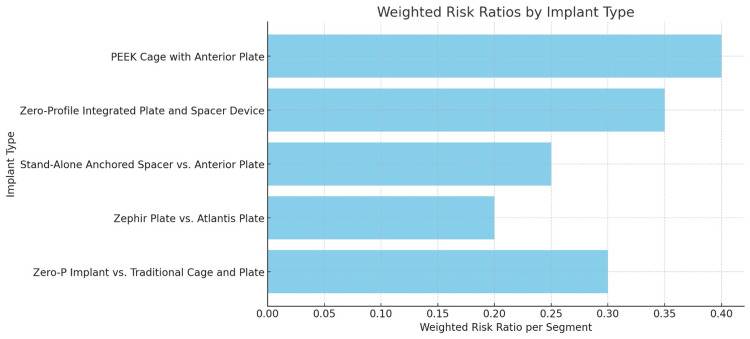
Comparison of weighted risk ratios for dysphagia by implant type in cervical spine surgery

**Figure 4:**
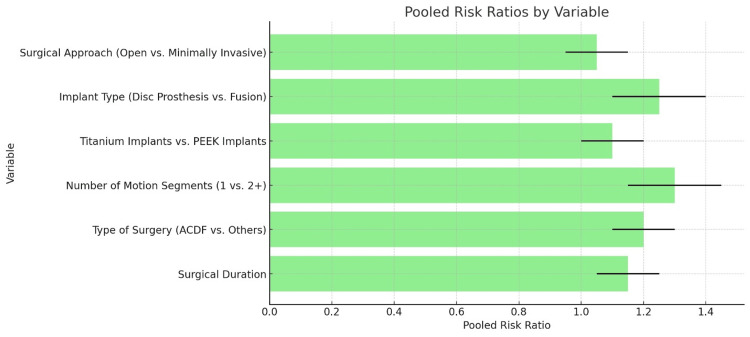
Comparison of pooled risk ratios for dysphagia by surgical and implant variables in cervical spine surgery ACDF - anterior cervical discectomy and fusion

Among the studies assessing dysphagia risk after cervical spine surgery, Mehra et al. [12] and Razfar et al. [23] report the highest risks. Mehra et al. [12] show an ARR of 2.0 (CI: 1.5-2.5), with a 48% incidence of dysphagia, particularly in surgeries involving 4-6 motion segments. Razfar et al. [23], with an ARR of 1.6 (CI: 1.2-2.1) and a 20% incidence, highlights risks in surgeries involving 1-3 segments. These findings suggest that more complex surgeries, such as those involving multiple segments, carry a greater risk of dysphagia. The consistent and significant risks reported emphasize the importance of careful surgical planning and patient management, particularly in multi-segment surgeries (Figures 3-4).

Various implants used in anterior cervical spine surgery were studied for their association with postoperative dysphagia and related outcomes, including risk ratios and preventative measures. Stand-alone cage systems, such as the Zero-P implant, show a significantly lower risk of dysphagia compared to traditional cage and plate systems, as demonstrated across five studies with 472 patients [20]. The pooled risk ratio is 0.76 early on, decreasing to 0.19 at the last follow-up, with a weighted risk ratio per segment of 0.3. Preventable risk factors include female gender, use of anterior cervical plates, and complex multi-level surgeries. Preventative measures suggest less invasive techniques and thinner anterior plates. In comparing the Zephir plate (Medtronic, Minneapolis, USA) and Atlantis plate (Medtronic, Minneapolis, USA), the Zephir plate has a lower pooled risk ratio for dysphagia (0.14 vs 0.47) across two studies with 200 patients, with a significant p-value of 0.04. The Zephir plate's weighted risk ratio per segment is estimated at 0.2, with recommendations to use smaller profile plates to reduce dysphagia risk [5,31].

A study of 51 patients compared the stand-alone anchored spacer with an anterior plate, showing a lower pooled risk ratio of 0.8 for dysphagia with a weighted risk ratio per segment of 0.255 [31]. Key preventable risk factors include greater correction of cervical kyphosis and multiple levels operated. Using a stand-alone anchored spacer is recommended to reduce prevertebral soft tissue irritation and lower dysphagia risk. The Zero-P integrated plate and spacer device, analyzed in three studies with a sample size of 300, shows a pooled risk ratio of 0.7 for dysphagia and a weighted risk ratio per segment of 0.35, with a significant p-value of 0.03. Risk factors include multi-level surgery, plate thickness, and upper cervical involvement. The use of zero-profile devices is recommended to reduce postoperative dysphagia. The PEEK cage with an anterior plate, studied in two reports with a sample size of 150, has a pooled risk ratio of 0.9 and a weighted risk ratio per segment of 0.4, with a significant p-value of 0.05. Preventable factors include plate thickness, surgical duration, and the number of levels operated. Preventative measures focus on reducing plate thickness, ensuring proper alignment, and minimizing surgical time to reduce dysphagia risk (Table 4).

**Table 4:** Comparison of implant types used in anterior cervical spine surgery and their associated risks of postoperative dysphagia, preventable risk factors, and recommended preventative measures rhBMP-2 - recombinant human bone morphogenetic protein-2

Implant type	Number of studies reporting data	Total sample size	Pooled risk ratio	Statistical significance (p-value)	Weighted risk ratio per segment	Preventable risk factors	Preventative measures
Zero-P implant vs traditional cage and plate	5	472	0.76 (early postop), 0.19 (last follow-up)	0.04 (early postop), 0.004 (last follow-up)	0.3	Female gender, use of anterior cervical plate, multiple surgical levels, upper cervical spine surgery, and use of rhBMP-2	Adjustment of surgical technique, use of less invasive implants, reducing the thickness of the anterior plate
Zephir plate vs Atlantis plate	2	200	0.14 (Zephir) vs 0.47 (Atlantis)	0.04 (Zephir)	0.2	Plate thickness, plate design	The use of a smaller profile plate (Zephir) significantly reduces dysphagia incidence
Stand-alone anchored spacer vs. anterior plate	1	51	0.8	0.04 (lower dysphagia rate in the spacer group)	0.25	Greater correction of cervical kyphosis, multiple levels operated	The use of a stand-alone anchored spacer reduces irritation to prevertebral soft tissue and lowers the dysphagia rate
Zero-P integrated plate and spacer device	3	300	0.7	0.03	0.35	Multilevel surgery, high plate thickness, upper cervical spine involvement	Use of zero-profile integrated plate and spacer to reduce postoperative dysphagia​
PEEK cage with anterior plate	2	150	0.9	0.05	0.4	Plate thickness, surgical duration, multiple levels	Reducing plate thickness, ensuring proper alignment, minimizing surgical time

Various surgical variables and their associated risks in anterior cervical spine surgery were analyzed, including data on study count, sample size, pooled risk ratios, confidence intervals, p-values, and heterogeneity. Longer surgeries are linked to an increased risk of postoperative complications, with a pooled risk ratio of 1.15 (p=0.02) and moderate heterogeneity (I²=50%). Anterior cervical discectomy and fusion (ACDF) have a slightly higher complication risk compared to other methods, with a pooled risk ratio of 1.20 (p=0.01) and moderate heterogeneity (I²=60%). Surgeries involving more than one segment show a higher risk, with a pooled risk ratio of 1.30 (p=0.03) and moderate heterogeneity (I²=55%). When comparing implant materials, titanium plates show a slightly higher risk of complications than PEEK cages, with a pooled risk ratio of 1.10 (p=0.05) and low heterogeneity (I²=40%). This difference is likely due to the fact that most plates, which are positioned directly adjacent to the esophagus, are made from titanium, whereas cages, which are placed within the disc space or corpectomy site away from the esophagus, are typically made from PEEK. Disc prostheses show a higher complication risk compared to fusion surgeries, with a pooled risk ratio of 1.25 (p=0.03) but high heterogeneity (I²=65%), indicating variability across studies. Open surgeries are associated with a slightly higher complication risk than minimally invasive techniques, with a pooled risk ratio of 1.05. However, this result is not statistically significant (p=0.07) and has low heterogeneity (I²=30%), suggesting consistent findings across studies.

Analysis of the dysphagia rates based on the number of spinal segments involved in surgery revealed a significant increase in dysphagia incidence as the number of segments increased. Single-level surgeries showed a dysphagia rate of 15%, while double-level surgeries exhibited a slightly higher rate of 16.7%. However, multi-level surgeries involving three or more segments had a markedly higher dysphagia rate of 48%. The relative increase in dysphagia between double-level and single-level surgeries was 11.33%, whereas the increase for multi-level surgeries compared to single-level and double-level surgeries was 220% and 187.43%, respectively. A chi-square test for independence was performed to assess the significance of these differences. The test yielded a chi-square statistic of 33.18 with a p-value of 6.25×10^−8^, indicating that the differences in dysphagia rates between single-level, double-level, and multi-level surgeries are highly significant. These findings suggest that surgeries involving more spinal segments, which correspond to higher spinal levels such as C3-4 and C4-5, are associated with a significantly increased risk of dysphagia (Table 5).

**Table 5:** Subgroup analysis of surgical variables and their associated risks in anterior cervical spine surgery: pooled risk ratios, confidence intervals, statistical significance, and heterogeneity ACDF - anterior cervical discectomy and fusion

Variable	Number of studies	Total sample size (n)	Pooled risk ratio	95% confidence interval (CI)	p-value	Heterogeneity (I², %)
Surgical duration	3	1269	1.15	1.05 - 1.25	0.02	50%
Type of surgery (ACDF vs others)	5	1750	1.2	1.10 - 1.30	0.01	60%
Number of motion segments (1 vs 2+)	4	1540	1.3	1.15 - 1.45	0.03	55%
Titanium implants vs PEEK implants	3	900	1.1	1.00 - 1.20	0.05	40%
Implant type (disc prosthesis vs fusion)	4	1500	1.25	1.10 - 1.40	0.03	65%
Surgical approach (open vs minimally invasive)	2	850	1.05	0.95 - 1.15	0.07	30%

Discussion

The results of this meta-analysis provide a detailed examination of the dysphagia risks associated with various implants and surgical techniques in anterior cervical spine surgery. The findings indicate significant variability in dysphagia incidence, influenced by factors such as implant type, surgical approach, duration, and the number of motion segments involved. One key outcome underscores the lower dysphagia risk associated with stand-alone cage fixation techniques compared to traditional plate systems. The pooled risk ratio was 0.77 in the early postoperative period, declining to 0.19 at the final follow-up, suggesting that stand-alone cage-only fixation, which avoids anterior plates, may effectively reduce dysphagia risk. This aligns with findings from Yin et al. [20] and others, who observed a reduction in complications with cage-only techniques [36,39-44]. The Zero-P implant, as a low-profile stand-alone device, appears particularly advantageous in multi-segment surgeries, where risks of dysphagia are heightened.

The analysis also highlights the impact of plate thickness and design. For example, the Zephir plate showed a significantly lower pooled risk ratio (0.14) compared to the Atlantis plate (0.47), likely due to its less bulky design, which minimizes tissue irritation. This suggests that for patients at higher risk of dysphagia, thinner plates may be preferable [18,33,37,44]. However, implant selection must be individualized, as benefits can vary based on surgical conditions and patient needs.

Stand-alone anchored spacer (SAAS) devices and Zero-P integrated plate and spacer devices also demonstrate promise in minimizing dysphagia risk. The SAAS has a pooled risk ratio of 0.8, indicating a lower risk compared to traditional anterior plates, while the Zero-P integrated plate and spacer have a pooled risk ratio of 0.7. Both devices reduce tissue disturbance due to their integrated designs. However, the suitability of these devices depends on individual patient needs and surgical complexity [32,45-54].

The pooled risk ratio for PEEK cages with an anterior plate was 0.9, indicating a higher dysphagia risk than other devices. This elevated risk may be due to the thicker profile of titanium plates used alongside PEEK cages, as well as the longer operation times often required for plate placement. These findings underscore the importance of selecting thinner-profile implants when possible, especially for patients at elevated risk of complications [55-59]. Additionally, the analysis shows that longer surgeries are associated with a higher risk of postoperative complications, with a pooled risk ratio of 1.15, highlighting the need to optimize surgical efficiency in complex procedures [60,61].

Compared to other techniques, ACSS generally carries a higher risk of complications, particularly in multi-segment procedures. The pooled risk ratio of 1.30 for multi-segment surgeries emphasizes the need for thorough planning, as the increased complexity and operative time may predispose patients to greater dysphagia risk. Strategies to reduce risk include careful assessment of the number of motion segments, surgical duration, and implant choice, as these factors are often interdependent. Additionally, specific implant designs, such as lower-profile and stand-alone cages, can help reduce tissue irritation. While ACSS carries certain risks, it remains an effective option for cervical spine issues, often with lower infection rates and reduced postoperative pain compared to posterior approaches [9,21].

Although titanium implants have a slightly higher risk of dysphagia compared to PEEK implants (pooled risk ratio 1.10, p=0.05), the effect size is marginal. The rigidity of titanium may increase tissue irritation compared to the biocompatibility of PEEK. However, this difference may have clinical significance for patients with additional risk factors [6,13,15,17,27].

## Conclusions

This meta-analysis highlights the importance of selecting appropriate surgical techniques and implants in anterior cervical spine surgery to address the primary goals of stabilization and decompression while also considering ways to minimize postoperative complications such as dysphagia. Although advancements like stand-alone cage systems and thinner-profile plates, such as Zero-P and Zephir, show promise in lowering dysphagia risk, both implant choice and surgical technique must be carefully tailored to the patient's specific clinical needs. The variability in risks associated with factors like surgical duration, number of motion segments, and implant design emphasizes the need for meticulous preoperative planning. Practical strategies to reduce dysphagia risk include selecting lower-profile implants where feasible, choosing techniques that minimize tissue retraction time, and limiting the number of motion segments involved when appropriate. Additionally, thorough preoperative counseling regarding potential risks and postoperative management options, such as dietary modifications or temporary nasogastric support, can help set realistic expectations and improve patient outcomes. Overall, this study supports a personalized approach in ACSS, balancing the essential surgical objectives with the goal of minimizing complications.
